# Supplementary material for: Role of the ADAM33 rs2280091 Variant in Modulating Lung Function in Cystic Fibrosis
Source: Int J Mol Sci. 2025 Nov 29;26(23):11583. doi: 10.3390/ijms262311583 (PMC12692732; doi:10.3390/ijms262311583)

Article

Role of the *ADAM33* rs2280091 Variant in Modulating Lung Function in Cystic Fibrosis

Vinícius Santiago dos Santos [1,2,3,†], Lucas Silva Mello [1,2,3], Luiz Felipe Azevedo Marques [1,2,3], Luana Rodrigues Silva [1,2,3], Carmen Sílvia Bertuzzo [4], José Dirceu Ribeiro [4] and Fernando Augusto Lima Marson [1,2,3,*,†]

[1] Laboratory of Molecular Biology and Genetics, Postgraduate Program of Health Sciences, Postgraduate Program of Health Data Science, University of São Francisco (Universidade São Francisco—USF), Bragança Paulista 12916-900, SP, Brazil; vinicius.santiago.santos@mail.usf.edu.br (V.S.d.S.); lucas.silva.mello@mail.usf.edu.br (L.S.M.); luiz.azevedo@mail.usf.edu.br (L.F.A.M.); luana.ssouza@mail.usf.edu.br (L.R.S.)
[2] Laboratory of Clinical and Molecular Microbiology, Postgraduate Program of Health Sciences, Postgraduate Program of Health Data Science, University of São Francisco (Universidade São Francisco—USF), Bragança Paulista 12916-900, SP, Brazil
[3] LunGuardian Research Group—Epidemiology of Respiratory and Infectious Diseases, Postgraduate Program of Health Sciences, Postgraduate Program of Health Data Science, University of São Francisco (Universidade São Francisco—USF), Bragança Paulista 12916-900, SP, Brazil
[4] Department of Pediatrics, University of Campinas (Universidade de Campinas—Unicamp), Campinas 13083-970, SP, Brazil; bertuzzo@unicamp.br (C.S.B.); jdirceuribeiro@gmail.com (J.D.R.)
* Correspondence: fernandolimamarson@hotmail.com or fernando.marson@usf.edu.br
† These authors contributed equally to this work.

Supplementary Figure S1

Supplementary Figure S1. Molecular and Clinical Aspects of Cystic Fibrosis (CF) and Genetic Modulation. A) Clinical manifestations of CF. CF is a multisystemic genetic disorder caused by mutations in the *CFTR* gene (Cystic Fibrosis Transmembrane Conductance Regulator), which encodes a protein responsible for *regulating* ion channels. In healthy individuals, this protein functions normally, allowing the production of mucus in small quantities, with low viscosity and minimal bacterial presence. In CF patients, the CFTR protein is altered, resulting in thick and abundant mucus that accumulates bacteria and inflammatory cells. This dysfunction affects several organs and systems, including the respiratory tract, where chronic rhinosinusitis, nasal congestion, discharge, and nasal polyposis are observed. The pancreas may develop exocrine insufficiency, while liver complications include cirrhosis, portal hypertension, and cholestasis. Intestinal manifestations such as distal intestinal obstruction syndrome, meconium ileus, and intussusception are common, and male infertility often results from congenital bilateral absence of the vas deferens. B) Functional classification of *CFTR* mutations. Mutations in the *CFTR* gene are grouped into six functional classes based on the type of defect they cause in the protein. Class IA mutations result in the absence of messenger RNA, while Class IB mutations lead to no CFTR protein production. Class II mutations impair intracellular trafficking of the protein, and Class III mutations affect channel gating. Class IV mutations reduce chloride and bicarbonate ion conductance, Class V mutations decrease protein synthesis, and Class VI mutations produce unstable CFTR proteins. These defects compromise ion transport across epithelial surfaces and contribute to disease severity. C) Genetic modulation of the CF phenotype. Beyond *CFTR*

mutations, variants in modifier genes can influence the clinical expression of cystic fibrosis. One example is the rs2280091 polymorphism in the *ADAM33* gene (A Disintegrin and Metalloproteinase 33), located on chromosome 20p13. This variant has been associated with bronchial hyperresponsiveness and persistent airway inflammation. Its presence may modulate the phenotype of CF patients who share the same *CFTR* genotype, highlighting the role of genetic background in shaping clinical variability.

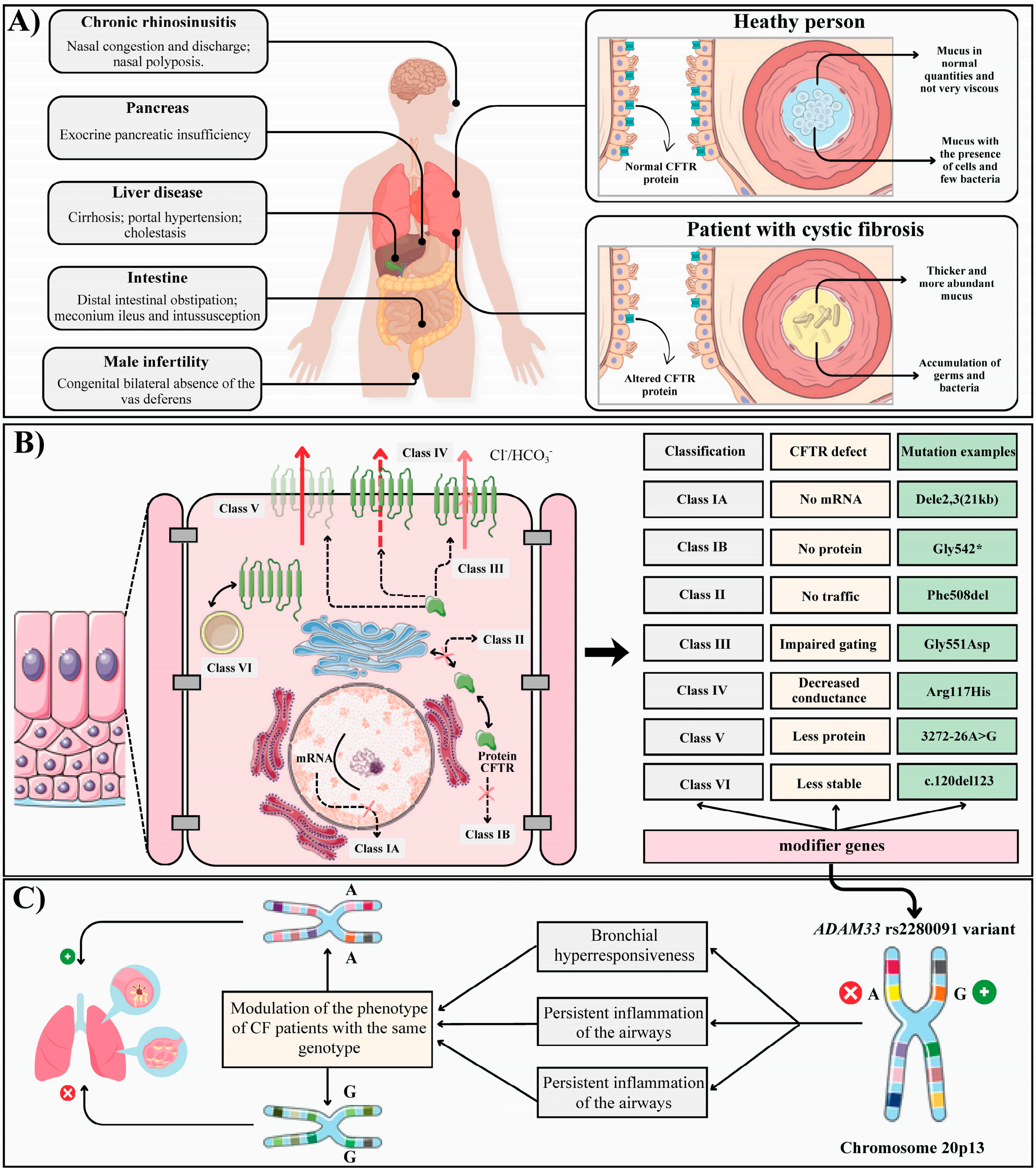

Supplement: Supplementary file 1 [file ijms-26-11583-s001.zip › ijms-3965293-supplementary.pdf]
